# Healthcare providers' knowledge and perceptions regarding the use of modern contraceptives among adolescent girls in Umlazi Township, KwaZulu-Natal province, South Africa

**DOI:** 10.11604/pamj.2021.38.124.20771

**Published:** 2021-02-04

**Authors:** Mbuzeleni Hlongwa, Boikhutso Tlou, Khumbulani Hlongwana

**Affiliations:** 1 Discipline of Public Health, School of Nursing and Public Health, University of KwaZulu-Natal, Durban, South Africa

**Keywords:** Contraceptive use, healthcare providers, knowledge, perceptions, adolescents

## Abstract

**Introduction:**

the phenomenon of unintended adolescent pregnancy continues to be a reproductive and public health concern in sub-Saharan Africa. Healthcare providers play an important role in influencing the use of contraceptives among adolescent girls. This study assessed knowledge and perceptions of healthcare providers regarding the use of modern contraceptives among adolescent girls in Umlazi township, KwaZulu-Natal province, South Africa.

**Methods:**

this was a descriptive study involving 35 healthcare providers covering all 10 primary healthcare clinics in Umlazi township. Data collected through a structured questionnaire were coded, entered into Epi data manager (version 4.6) and exported to STATA (version 15.0) for analysis.

**Results:**

of the thirty-five healthcare providers that participated in this study, professional nurses (54.3%) and enrolled nurses (17.1%) constituted the majority. The mean age of the participants was 42.11 years, with 88.6% being females. More than a third (37.1%) of healthcare providers did not know whether or not modern contraceptives make users promiscuous, while more than half (57%) had negative attitudes towards adolescents exploring contraceptive methods. Healthcare providers viewed health systems challenges, such as poor working conditions, long queues, and contraceptives stock-outs, as deterrents towards the provision of quality sexual behaviour counselling and modern contraceptive education to users.

**Conclusion:**

poor health systems and negative behaviours by healthcare providers influences the delivery of family planning services in primary healthcare clinics and serve as barriers to quality family planning services provided to younger women.

## Introduction

Unintended pregnancy continues to be a reproductive and public health concern in sub-Saharan Africa [1]. However, the extent to which women manage various aspects of their sexual and reproductive health, including the prevention of unplanned or unwanted pregnancies and exposure to HIV/AIDS, raises questions of public health and health promotion concern [2]. While the sub-Saharan African region experiences more than 14 million abortions related to unintended or unwanted pregnancies each year [3], adolescent women are the most affected by high unintended pregnancies and termination of pregnancies [4]. South Africa continues to experience a high number of termination of pregnancies [5], an indication that many pregnancies are unintended. Factors influencing poor modern contraceptive use among adolescent girls usually include stigma, embarrassment, and shame [6]. Negative attitudes of healthcare providers towards younger women have been reported to prevent adolescents from utilising contraceptive methods from local clinics [7,8]. Some healthcare providers have been reported to be hesitant or unprepared to provide contraceptive methods to younger women [9]. Instead, some healthcare providers would opt to deter adolescents from engaging in early sex [10]. Healthcare providers sometimes view the provision of modern contraceptives to unmarried adolescents girls as promoting sexual promiscuity [11]. Healthcare providers´ poor knowledge of different contraceptive methods has been flagged as one of the contributing factors to incomplete information sharing towards current and potential contraceptive users [12].

Less than half of the healthcare providers have been reported to have sufficient information about all the types of contraceptive methods available in clinic settings [13], which is an indication of lack of training or education regarding contraceptive methods accessible in primary healthcare clinics. While this may be indicative of poor training on contraceptives, this could also be related to their attitudes towards the contraceptives, which in turn would adversely affect their learning desire. In South Africa, lack of confidence was noted to adversely affect nurses effectively providing Implanon contraceptive method services [14]. While negative views towards some contraceptive methods persist among healthcare providers [14], age and length in practice have been shown to influence the prescribing patterns of contraceptives for adolescents. The recently graduated and/or younger healthcare providers are more likely to offer contraceptives to adolescents freely compared to their older counterparts [15]. In a province like KwaZulu-Natal, where unintended and termination of pregnancies are high, it is important to understand healthcare providers´ knowledge of modern contraceptive methods and their perceptions towards adolescents seeking modern contraceptive methods in primary healthcare clinics. This is an important step, in so far as, determining challenges related to adolescents accessing family planning services in primary healthcare clinics, is concerned. Therefore, this study aimed to assess knowledge and perceptions of healthcare providers regarding modern contraceptive use among adolescent girls in Umlazi Township, KwaZulu-Natal province, South Africa.

## Methods

**Study setting:** Umlazi township, is located in the province of KwaZulu-Natal, with an estimated population of more than half a million people [16]. The township falls under the Ethekwini metro, which has the highest HIV prevalence in South Africa [17]. Umlazi has 10 primary healthcare clinics and one hospital. All 10 clinics in Umlazi participated in the study. All 10 clinics combined serve more than 50 000 clients on average per month.

**Study design, participants and sampling:** a cross-sectional descriptive survey was conducted from November 2018 to April 2019. Of the 124 invited nurses, 108 agreed to participate in the study (87.1%), with only 35 returning the completed questionnaires (32.4%). Evidence shows that studies with nurses as participants often report lower response rates [18]. The study sample was drawn from healthcare providers covering all 10 Umlazi township´s primary healthcare clinics. All nurses who were available and providing health services at the time of data collection were approached, introduced to the study and invited to participate. Participants were only enrolled in the study after the health services had been rendered to clients.

**Study instrument and data collection:** a structured self-administered questionnaire was designed in English and a local language, IsiZulu. The trained and experienced research assistants, who understand both languages were available to clarify aspects of the questionnaire when required by the participants. The study instrument covered these aspects: demographic and socioeconomic characteristics of the study participants, awareness of modern contraceptives, capacity building, the use of modern contraceptives, the technical expertise, and information related to working conditions. Data quality assurance was achieved through data validation, data cleaning, questionnaire verification, as well as ensuring that questionnaires were field-tested for consistency. Administered questionnaires were initially checked by the principal investigator to ensure quality assurance of collected data and completeness of questionnaires.

**Data analysis:** data were coded, entered into Epi data manager (version 4.6) [19] and exported to STATA (version 15.0) [20] for analysis. Data cleaning was conducted to eliminate discrepancies in data before analysis were carried out. The background information of participants were analysed using descriptive statistics. The frequency distribution and cross-tabulations of each variable were carried out for categorical data. Frequency distributions of continuous variables were tested for normality using Shapiro-Wilks test.

## Results

**Demographic characteristics of the participants:** professional nurses (54.3%) and enrolled nurses (17.1%) constituted the most participants (Table 1). The ages of participants ranged between 26 to 63 years, with the mean age and the standard deviation of 42.11 and 11, respectively. Most healthcare providers were females (88.6%), and a large proportion of healthcare providers´ (80%) first language was IsiZulu. Married, single and divorced participants made up 34.3%, 28.6% and 17.1%, respectively.

**Table 1:** characteristics of healthcare providers (n=35)

Variable	Frequency	Percentage (%)
**Type of provider**		
Enrolled Nursing Assistant	3	8.6
Enrolled Nurse	6	17.1
Professional Nurse	19	54.3
No response	7	20.0
**Sex**		
Female	31	88.6
Male	4	11.4
**Home language**		
IsiZulu	27	80.0
English	3	8.6
Xhosa	3	8.6
Sotho	1	2.9
**Racial group**		
Black/African	32	91.4
Coloured	1	2.9
Asian/Indian	2	5.7
**Marital status**		
Married	12	34.3
Never married/single	10	28.6
Divorced	6	17.1
Widowed	3	8.6
No response	4	11.4
**Age:** ranged between 26-63 years		
Mean (SD): 42.11 (11)		

**Healthcare providers´ knowledge of modern contraceptive methods:** about a quarter (25.7%) of healthcare providers reported that modern contraceptives make users promiscuous, while 37.1% did not know (Table 2). More than half (57.2%) of participants indicated that modern contraceptives are the most effective way of avoiding or delaying pregnancies, while 11.5% was not so positive about the effectiveness of modern contraceptives. Over one-third (34.3%) of participants indicated that they have not had any form of training or refresher course on any contraceptive methods in the past 12 months. The majority of participants (74.3%) reported that they had not attended any training or refresher course on sexual behaviour and modern contraceptive use counselling in the past 12 months.

**Table 2:** healthcare providers' knowledge of modern contraceptive methods

Variable	Frequency	Percentage (%)
**Contraceptives make users promiscuous**		
No	7	20
Yes	9	25.7
Don't know	13	37.1
No response	6	17.1
**Contraceptives are harmful**		
No	23	65.7
Yes	3	8.6
Don't know	5	14.3
No response	4	11.4
**Contraceptives have side effects**		
No	2	5.7
Yes	25	71.4
Don't know	3	8.6
No response	5	14.3
**How often do contraceptives actually effective in planning families?**		
Never	3	8.6
Seldom	1	2.9
Sometimes	4	11.4
Most often	8	22.9
Always	12	34.3
No response	7	20.0
**Which contraceptive methods have you been trained on to give better guidance and education to your clients in the past 12 months?**		
Condom	3	8.6
Injections	6	17.1
More than 1 contraceptives	9	25.7
None	12	34.3
No response	5	14.3
**Have you been trained or given refresher training on sexual behaviour counselling in the past 12 months?**		
No	26	74.3
Yes	4	11.4
No response	5	14.3
**Have you been trained or given refresher training on contraceptive use counselling in the past 12 months?**		
No	26	74.3
Yes	5	14.3
No response	4	11.4
**Have you been trained or given refresher training on sexual and reproductive health education in the past 12 months?**		
No	26	74.3
Yes	4	11.4
No response	5	14.3

**Healthcare providers´ perceptions towards modern contraceptive use and users:** the majority (74.3%) of participants reported that modern contraceptive users have good knowledge of different contraceptive methods, with 68.6% reporting that users understand the different modern contraceptive methods available in Umlazi township primary healthcare clinics (Table 3). More than half (51.4%) of participants reported that users are aware of side effects from using particular contraceptive methods, with 20% reporting the opposite. The majority (80.0%) of participants disagreed that healthcare providers do provide sexual health behaviour counselling and education each time a woman visit the clinic. The majority (80.0%) of participants disagreed that healthcare providers are friendly and open despite a woman´s age and HIV status when seeking modern contraceptives. Most (82.9%) participants reported that healthcare providers do judge women when acquiring services, and 77.1% disagreed that women do not feel judged at all by healthcare providers. More than half (57.2%) of participants reported that they either mostly or always advise adolescents to abstain from sex when they seek modern contraceptives. The majority (91.4%) of participants reported that women have a good to excellent attitude when acquiring contraceptive methods in Umlazi township clinics.

**Table 3:** healthcare providers' perceptions of modern contraceptive use and users

Variable	Frequency	Percentage (%)
**Do you believe that your clients understand the different contraceptive methods available to them from this facility?**		
No	5	14.3
Yes	24	68.6
Don't know	2	5.7
Not answered	4	11.4
**Do you think your clients know the side effects of using particular contraceptives?**		
No	7	20.0
Yes	18	51.4
Don't know	4	11.4
Not answered	6	17.1
**Nurses at this clinic provide sexual health behaviour counselling and education each time a woman visits the clinic**		
Strongly agree	1	2.9
Neither agree nor disagree	3	8.6
Moderately disagree	7	20.0
Strongly disagree	21	60.0
Not answered	3	8.6
**Nurses at this clinic are friendly and open despite a woman's age and HIV status when acquiring family planning services**		
Strongly agree	2	5.7
Moderately disagree	6	17.1
Strongly disagree	22	62.9
Not answered	5	14.3
**Nurses at this clinic do not judge women at all despite the service provided**		
Strongly agree	2	5.7
Moderately disagree	7	20.0
Strongly disagree	22	62.9
Not answered	4	11.4
**Do you advise adolescents to abstain from sex when they seek contraceptives?**		
No	8	22.9
Rare times	3	8.6
Most of the times	12	34.3
Always	8	22.9
Not answered	4	11.4
**How is the attitude of women when acquiring contraception in the clinic?**		
Excellent	9	25.7
Very Good	7	20.0
Good	16	45.7
Fair	1	2.9
Poor	1	2.9
Not answered	1	2.9

**Healthcare providers´ observation of modern contraceptive usage, including how they advise clients:** nearly half (45.7%) of participants reported that they generally recommend injectable contraceptives to their clients (Table 4). The majority (80.0%) of participants reported that the intrauterine contraceptive device (IUD) is the most commonly requested contraceptive method by users. Some concerns were raised regarding the modern contraceptives, such as injectable (28.6%), the pill contraceptives (28.6%), Implanon (14.3%) and the IUD (14.3%). Concerns regarding side-effects were reported by 71.4% participants, whereas 54.3% of the participants were concerned about the unbearable waiting hours and clinic conditions. The majority (74.3%) of users disagreed that women´s modern contraceptive methods of choice are always available in Umlazi township clinics. More than half (60.0%) of participants disagreed that the working environment in clinics is conducive enough to provide sexual behaviour and contraceptive use counselling and education to clients.

**Table 4:** healthcare providers' observation of modern contraceptive usage

Variable	Frequency	Percentage (%)
**Which contraceptive method do you generally recommend to your clients?**		
Injections	16	45.7
IUD	7	20.0
Condom	5	14.3
Abstinence	1	2.9
More than 1 contraceptives	3	8.6
No response	3	8.6
**What is the most requested contraceptive method by your clients?**		
Injections	2	5.7
IUD	28	80.0
Condom	1	2.9
No response	4	11.4[MH1]
**Which contraceptive method do your clients complain about the most?**		
Condom	3	8.6
IUD	5	14.3
Injections	10	28.6
Implanon	5	14.3
Pill	10	28.6
None	1	2.9
No response	1	2.9
**What do clients normally complain about regarding those contraceptives?**		
Side effects	25	71.4
Other	4	11.4
Not applicable	3	8.6
Not answered	3	8.6
**Working conditions/health systems**		
**The waiting hours are unbearable at this clinic**		
Strongly agree	13	37.1
Moderately agree	6	17.1
Neither agree nor disagree	6	17.1
Moderately disagree	4	11.4
Strongly disagree	2	5.7
Not answered	4	11.4
**Women's contraception of choice always available in this clinic**		
Strongly agree	5	14.3
Neither agree nor disagree	1	2.9
Moderately disagree	9	25.7
Strongly disagree	17	48.6
Not answered	3	8.6
**The working environment is conducive enough to provide sexual behaviour and contraceptive use counselling and education to clients**		
Strongly agree	2	5.7
Moderately agree	3	8.6
Neither agree nor disagree	5	14.3
Moderately disagree	5	14.3
Strongly disagree	16	45.7
Not answered	4	11.4

## Discussion

Our findings revealed a gap in contraceptive-related training programmes aimed at benefiting clients seeking contraception services in primary healthcare clinics in Umlazi township. Healthcare providers´ knowledge raises serious concerns, given the high number of healthcare providers who do not know whether or not modern contraceptives make users promiscuous (37.1%) and those (25.7%) who believed that modern contraceptives make users promiscuous. This is consistent with literature, such as a study conducted in Nigeria where 57% of nurses reported that providing contraceptives to unmarried adolescents promotes sexual promiscuity [11]. The notion, particularly held by men, that using modern contraceptives encourages adolescent girls to become sexually promiscuous was also reported in Kenya and should be corrected [21]. Lack of training remains an obvious barrier to the provision of quality family planning services [22]. This is consistent with the findings of the study conducted in similar setting, whereby healthcare providers showed poor understanding of different modern contraceptive methods [9]. Nurses can also influence the choice of contraceptive method. The quality of counselling may motivate or serve as a barrier to adolescent girls accessing modern contraceptives. Arising from poor knowledge and/or unavailability of contraceptive methods, healthcare providers often limit contraceptive methods discussions to few methods [23]. Discouragement of adolescent girls from using contraceptives by healthcare providers was one of the striking findings worthy of targeted intervention. Healthcare providers appeared to be generally comfortable with advising adolescents to abstain from sex as evidenced in the studies conducted in South Africa, Kenya, and Zambia [10,24].

This is despite a wide body of evidence revealing that the majority of adolescents in sub-Saharan Africa and South Africa, in particular, do engage in sex [25, 26]. This provider attitude may likely discourage younger women from seeking contraceptive methods in primary healthcare clinics, for fear of being judged and/ or dissuaded from receiving their preferred contraceptive method. Healthcare providers in this study had negative attitudes towards women´s age, as well as judgemental towards women when acquiring family planning services. This is concerning and directly contravenes women´s sexual and reproductive health rights as prescribed by the World Health Organization [27,28]. The South African guidelines on contraception also emphasize the need for expanding contraceptive choices to all women, despite their age and HIV status [23]. Concerns over age restrictions on contraceptive provision have also been reported in similar settings [7,9] and continue to occupy public discourse. This study further found that health systems challenges, such as poor working conditions, long waiting queues, and contraceptives stock-outs may compromise the provision of quality contraception services. The results of this study are consistent with other studies conducted in South Africa, where it was noted that non-availability of contraceptive methods, long waiting hours and nurses´ negative attitudes at clinics are likely to be negatively associated with contraceptive use [29,30]. More than 60% of women reported the unavailability of contraceptives as the reason for poor usage of contraception in South Africa [31]. In South Africa, primary healthcare clinics are known for being understaffed and overloaded with patients, thereby resulting in patients waiting long periods of time (hours) in queues, which in turn may likely discourage women from asking family planning-related questions [30,32,33].

While this study provides an important contribution in the field of sexual and reproductive health, it has notable limitations. The study sample size was small (n=35) and confined to Umlazi township primary healthcare clinics, an indication that healthcare providers were under-represented in the sample. This limits the generalisability of the findings. Due to high workloads and long queues nurses face each day, this small sample size can be justified. Only nurses available at the clinic during the time of data collection were included (convenience). Therefore, the findings of this study should be interpreted with caution. Nevertheless, the participants of this study represented all the 10 primary healthcare clinics in Umlazi township. Therefore, the insights gained from the participants have a potential for use in other similar settings in South Africa. Conducting similar studies on a larger scale, with a bigger sample size and extended geographical area is essential for a broader understanding of all the issues affecting the contraceptive use by adolescent girls in South Africa, against the backdrop of high teenage pregnancy compounded with high HIV prevalence. Understanding healthcare providers´ knowledge and perceptions related to family planning services are important for designing targeted interventions aimed at improving contraceptive uptake by adolescent girls. There is a need for a provider retraining or refresher courses on various contraceptive methods and sexual and reproductive health education. Strategies to strengthen healthcare providers´ knowledge and skills about different contraceptive methods, as well as changing restrictive attitudes towards younger or HIV positive women are key to addressing unintended pregnancies. This also has the potential for improving contraceptive access to women. Educational programmes about contraceptive methods, benefits, uses, and side effects should be strengthened and implemented at community levels, as well as hospitals and clinic waiting rooms. Healthcare systems should be strengthened to meet the contraceptive demands and addressing issues related to long queues. While South Africa is implementing the provision of integrated healthcare services, it is important that this initiative is strengthened to improve and facilitate access to family planning services.

## Conclusion

The findings of this study revealed that healthcare providers’ attitudes may negatively affect younger women from seeking and accessing contraceptive methods in primary healthcare clinics. Health systems challenges, such as long queues and poor working conditions and shortages of contraceptive methods should be addressed in order to improve the quality of family planning services provided to younger women. Initiatives aimed at improving healthcare providers’ knowledge of contraceptive methods and attitudes towards younger women should be prioritized

### What is known about this topic

- Although nurses’ perceptions on contraceptive use among young women is documented elsewhere, understanding this in a setting with high unintended pregnancies, termination of pregnancies, HIV/AIDS, risky sexual behaviors is important;
- Perceptions of healthcare providers remain critical aspects of whether or not younger women seek contraceptive use in primary healthcare clinics.

### What this study adds

- Key information on healthcare providers’ knowledge and perceptions related to family planning services towards adolescents in a setting with high unintended pregnancies;
- Understanding of healthcare systems challenges that may prevent adolescent girls from accessing contraceptive methods.
